# Single-cell RNA sequencing analysis to explore immune cell heterogeneity and novel biomarkers for the prognosis of lung adenocarcinoma

**DOI:** 10.3389/fgene.2022.975542

**Published:** 2022-08-15

**Authors:** Yong Xu, Yao Wang, Leilei Liang, Nan Song

**Affiliations:** Department of Thoracic Surgery, Shanghai Pulmonary Hospital, School of Medicine, Tongji University, Shanghai, China

**Keywords:** lung adenocarcinoma, single-cell sequencing, tumor heterogeneity, tumor immunity, prognosis

## Abstract

**Background:** Single-cell RNA sequencing is necessary to understand tumor heterogeneity, and the cell type heterogeneity of lung adenocarcinoma (LUAD) has not been fully studied.

**Method:** We first reduced the dimensionality of the GSE149655 single-cell data. Then, we statistically analysed the subpopulations obtained by cell annotation to find the subpopulations highly enriched in tumor tissues. Monocle was used to predict the development trajectory of five subpopulations; beam was used to find the regulatory genes of five branches; qval was used to screen the key genes; and cellchart was used to analyse cell communication. Next, we used the differentially expressed genes of TCGA-LUAD to screen for overlapping genes and established a prognostic risk model through univariate and multivariate analyses. To identify the independence of the model in clinical application, univariate and multivariate Cox regression were used to analyse the relevant HR, 95% CI of HR and *p* value. Finally, the novel biomarker genes were verified by qPCR and immunohistochemistry.

**Results:** The single-cell dataset GSE149655 was subjected to quality control, filtration and dimensionality reduction. Finally, 23 subpopulations were screened, and 11-cell subgroups were annotated in 23 subpopulations. Through the statistical analysis of 11 subgroups, five important subgroups were selected, including lung epithelial cells, macrophages, neuroendocrine cells, secret cells and T cells. From the analysis of cell trajectory and cell communication, it is found that the interaction of five subpopulations is very complex and that the communication between them is dense. We believe that these five subpopulations play a very important role in the occurrence and development of LUAD. Downloading the TCGA data, we screened the marker genes of these five subpopulations, which are also the differentially expressed genes in tumorigenesis, with a total of 462 genes, and constructed 10 gene prognostic risk models based on related genes. The 10-gene signature has strong robustness and can achieve stable prediction efficiency in datasets from different platforms. Two new molecular markers related to LUAD, HLA-DRB5 and CCDC50, were verified by qPCR and immunohistochemistry. The results showed that *HLA-DRB5* expression was negatively correlated with the risk of LUAD, and CCDC50 expression was positively correlated with the risk of LUAD.

**Conclusion:** Therefore, we identified a prognostic risk model including CCL20, CP, HLA-DRB5, RHOV, CYP4B1, BASP1, ACSL4, GNG7, CCDC50 and SPATS2 as risk biomarkers and verified their predictive value for the prognosis of LUAD, which could serve as a new therapeutic target.

## Introduction

Lung cancer is still one of the main types of cancer, and its mortality is still the highest of all cancers (Siegel et al., 2021). Lung adenocarcinoma (LUAD) is the most common histological subtype of lung cancer, accounting for almost half of all lung cancer deaths (Kenfield et al., 2008; Houston et al., 2014). Because of the decline in smoking rates, the incidence and mortality of many other types of lung cancer, such as squamous cell lung cancer and small cell lung cancer, have been decreasing. The incidence rate and incidence rate of LUAD are increasing (Remen et al., 2018; Barta et al., 2019; Choi et al., 2019; Tseng et al., 2019). At present, the treatment of patients with advanced LUAD is still limited to targeted therapy and radiotherapy and chemotherapy, and the prognosis is still very poor. Therefore, finding accurate prognostic biomarkers and effective therapeutic targets is still of great significance to improve the poor prognosis of LUAD patients.

In recent decades, high-throughput sequencing technology has been widely used in various fields of biology and medicine, which has greatly promoted related research and applications. However, traditional transcriptome sequencing technology (bulkRNA-seq) is based on tissue samples or cell populations, which reflect the average expression level of genes in the cell population, but there is extensive heterogeneity between cells, which is of great significance for targeted therapy of tumors (Dagogo-Jack and Shaw, 2018). In recent years, single-cell RNA SEQ (scRNA-seq) technology has developed vigorously. ScRNA-seq can reveal the expression of all genes in the whole genome at the single-cell level and study cell heterogeneity more intuitively (Lavin et al., 2017). At present, scRNA-seq has been widely used in different types of tissues and cell lines of various species (especially human and mouse), including normal and diseased cells. Single-cell sequencing has been used in the study of pancreatic cancer, colon cancer, and so on, but (Moncada et al., 2020; Zhang L. et al., 2020; Liang et al., 2021) it has not been widely studied in lung cancer. We found and defined the cell subsets of LUAD by single-cell analysis and explored their predictive ability in the prognosis of LUAD.

This study screened cell types with significant differences in subpopulation abundance through single-cell analysis and screened cell types with different subpopulation abundance. At the same time, combined with LUAD bulkRNA-seq in TCGA data, the marker genes related to prognosis were screened, and the risk model was constructed accordingly. Finally, we identified a prognostic risk model and verified its predictive value for the prognosis of LUAD, which could serve as a new therapeutic target.

## Materials and methods

### Data acquisition and preprocessing

The single-cell sequencing data GSE149655 were downloaded from the GEO database. A total of four samples were detected, including two LUAD samples and two normal samples. The bulkRNA-seq data of LUAD were downloaded from the TCGA database and further processed and transformed into TPM data.

The clinical phenotype data of TCGA-LUAD were downloaded, and the samples lacking survival time and survival status were eliminated. GSE31210 of LUAD was downloaded from the GEO database. By transforming the probe into a gene symbol, multiple gene symbols corresponding to one probe were removed, and the average value of one symbol corresponding to the probe was taken.

### Clustering dimensionality reduction of single-cell data

First, the single-cell data were filtered by setting each gene to be expressed in at least three cells and each cell to express at least 250 genes, calculating the proportion of mitochondria and rRNA through the percentagefeatureset function, and ensuring that the gene expressed by each cell was greater than 500 and the mitochondrial content was less than 35%. Then, we counted the number of cells in each sample before and after filtration. Then, the merged data are standardized through log normalization. Find highly variable genes through the findvariablefeatures function (identify variable characteristics based on variance stabilization transformation (“VST”) and then scale all genes by using the scaledata function and PCA dimensionality reduction to find anchor points. We selected dim = 40 and clustered the cells through the findneighbors and findclusters functions (set resolution = 0.5).

### Subgroup definition

We downloaded the marker genes of human cells from the official website of CellMarker (http://biocc.hrbmu.edu.cn/CellMarker/). At the same time, the corresponding subgroups of cluster profilers are selected through the corresponding functions of cluster profilers.

### Subgroup statistical analysis

A total of 11 subpopulations were obtained through cell annotation. We counted the number of cells in tumor samples and normal samples, constructed a 2 * 2 contingency table, calculated the *p* value using Fisher’s test (bilateral test), and calculated the corresponding difference multiple (TVSN). To define the development trajectories of the five cell subsets, we used Monocle to predict the development trajectories of the five subsets. Then, we used the BEAM (branched expression analysis modelling) method to find the regulatory genes of five branches, screened the key genes with qval (corrected P), screened the 100 genes with the smallest qval, drew the heatmap and enriched the pathway.

### Screening of key genes and construction, evaluation and validation of the prognostic risk model

The expression profile data of FPKM of TCGA were downloaded and further transformed into TPM. The standard deviation of each gene expressed in all samples was greater than 0.5 for filtering. The expression profile matrix of LUAD was analysed by the limma package, and the differentially expressed genes were screened by | logfc | > 1 and FDR <0.05.

First, single-factor risk analysis is carried out. Using the expression profile data of TCGA, for the related genes and survival data, the univariate Cox proportional hazards regression model was carried out by using the R-package survival Cox function, and *p* < 0.01 was selected as the threshold for filtering.

Next, multivariate analysis is carried out. Lasso regression was used to further compress the genes to reduce the number of genes in the risk model. Next, 10-fold cross validation was used to build the model and analyse the confidence interval under each lambda.

The risk score of each sample was calculated according to the expression level of the sample, and the risk score distribution of the sample was drawn. ROC analysis of the prognostic classification of the risk score was carried out by using the R software package timeROC, and the prognostic prediction and classification efficiency at 1, 3 and 5 years were analysed. Finally, we calculated the z score for the risk score, divided the samples with risk scores greater than zero into a high-risk group and a low-risk group, and drew a KM curve. Lasso regression and the risk score were performed as previously described (Yu et al., 2021).

Finally, we used the GEO dataset (GSE31210) to verify the model.

Univariate and multivariate analysis of the 10-gene signature and its relationship with pathways.

To identify the independence of the 10-gene signature model in clinical application, the relevant HR, 95% CI of HR and *p* value were analysed by univariate and multivariate Cox regression in the clinical information carried by all TCGA data. The clinical information recorded by TCGA patients was systematically analysed, including sex, stage and risk type.

To further observe the relationship between the risk scores of different samples and biological functions, the expression profiles corresponding to TCGA samples were analysed by single-sample GSEA (ssGSEA) with the R software package GSVA, and the scores of each sample on different functions were calculated; that is, the ssGSEA scores of each sample corresponding to each function were obtained, and the correlation between these functions and risk scores was further calculated. A function with a correlation of no less than 0.3 was selected.

### Tissue samples

Samples of LUAD and normal tissues were collected from 15 patients (all >16 years of age), immediately placed in liquid nitrogen and preserved at −80°C. None of the LUAD patients received preoperative antitumor therapies. Patients and their families in this study were fully informed, and informed consent was obtained from all participants. This study was approved by the Ethics Committee of Shanghai Pulmonary Hospital (K20-148Y).

### RNA isolation and quantitative real-time PCR

Briefly, total RNA was isolated from tissues and cells by using TRIzol^®^ reagent (Thermo Fisher Scientific, Inc.) and then reverse transcribed using a QuantiTect Reverse Transcription Kit (QIAGEN, Valencia, CA) according to the manufacturer’s specifications. qPCR amplification was performed by using SYBR-Green PCR mix (Takara), and the expression levels of target genes were normalized to the level of GAPDH. The primer sequences were as follows: HLA-DRB5 Forward Sequence-GAACAGCCAGAAGGACTTCCTG and Reverse Sequence-GCAGGATACACAGTCACCTTAGG. CCDC50 Forward Sequence-AGTGATGAACCTCACCATTCTAAG and Reverse Sequence-GAAATGCCGTGTGGAACTCTGC.

### Immunohistochemistry

Each group of sarcoma samples was fixed in 10% formalin, embedded in paraffin, and processed into 5 µm continuous sections. Samples were incubated in rabbit anti-HLA-DRB5 (Origene, OTI4G7; 1:1,200) anti-CCDC50 (Abcam, ab127169; 1:1,200) overnight at 4°C, followed by incubation with horseradish peroxidase-coupled goat anti-rabbit secondary antibody at 37°C for 30 min. The experimental procedure was performed according to strict adherence to the manufacturers’ instructions. The IHC quantitation analysis was calculated by ImageJ software.

## Results

### Screening and definition of single-cell subsets of LUAD

First, the genes were screened. Supplementary Figure S1 is the quality control chart before filtration, and Supplementary Figure S2 is the quality control chart after filtration. Then, we counted the number of cells in each sample before and after filtration, as shown in Supplementary Figure S3A.

Supplementary Figure S3B (left) shows the distribution of hypervariable genes and non-hypervariable genes, and the top 20 hypervariable genes are shown in Supplementary Figure S3B (right).

**FIGURE 1:**
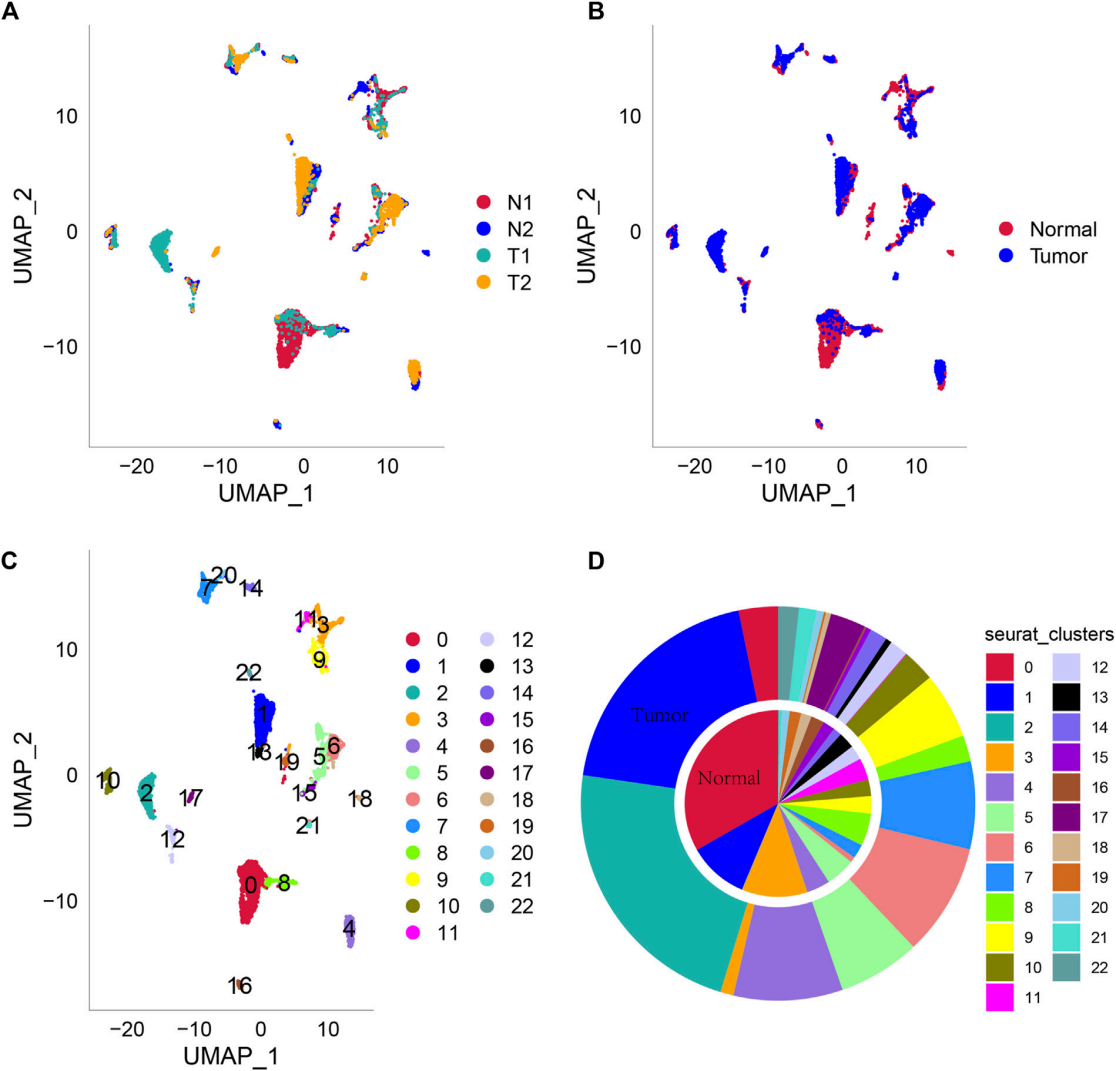
Umap dimensionality reduction. **(A)** Cell distribution map of four tissue samples. **(B)** Cell distribution map of tumor tissue and normal tissue samples. **(C)** Cell distribution map of 23 subpopulations. **(D)** The abundance of each subgroup of the two tissue types is normal and Turkish from inside to outside.

**FIGURE 2:**
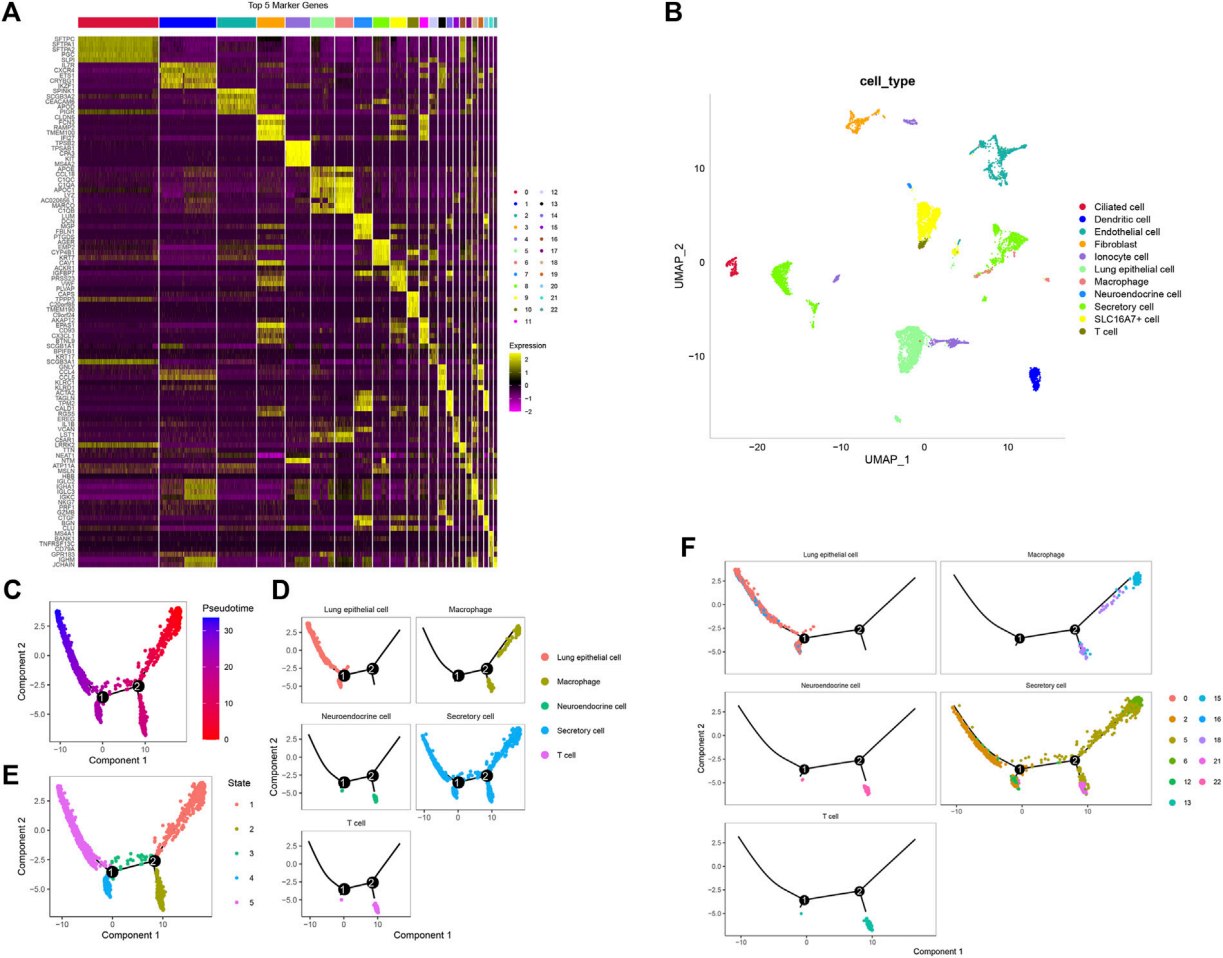
The findallmarkers function to screen marker genes. **(A)** Expression of the top 5 marker genes in 23 subpopulations. **(B)** Umap diagram of the distribution of 11 subpopulations. **(C)** Pseudotime measures the degree of cell differentiation. **(D)** The five subgroups can be divided into five branches. **(E)** Track of differentiation of the five subgroups. **(F)** Cell trajectories of five different types of subpopulations.

Then, all genes were scaled by using the scaledata function, and anchor points were found by PCA dimensionality reduction (Supplementary Figure S3C). Next, the cells were clustered, and 23 subpopulations were obtained. Then, we select the first 40 PCs and use umap to further reduce the dimension. The distribution of the four samples is shown in Figure 1A. Two of the four samples were tumor tissues, and two were normal tissues. The distribution of cells in tumor tissues and normal tissues is shown in Figure 1B, and Figure 1C shows the distribution of 23 cell subsets. At the same time, we counted the abundance of these 23 subpopulations in each sample (Figure 1D). Next, we used the findallmarkers function to screen marker genes of 23 subgroups by logfc = 0.5 (differential multiple), minpct = 0.3 (minimum expression ratio of differential genes) and screened them with corrected *p* < 0.05. Here, we only show the expression of the top 5 marker genes with the most prominent contribution in each subgroup (Figure 2A). The results of marker genes are shown in Supplementary Table S1.

We downloaded the marker genes of human cells from the official website of CellMarker (http://biocc.hrbmu.edu.cn/CellMarker/), selected the corresponding organization of the lung, and Supplementary Table S2 was the list of marker genes of cells. At the same time, through the enricher function of the clusterprofiler package, the definition of 23 subsets is finally completed. As shown in Table 1, 23 clusters are annotated to 11 subgroups. By merging these subgroups, 11 subgroups (ciliated cells, dendritic cells, endothelial cells, fibroblasts, monocyte cells, lung epithelial cells, macrophages, neuroendocrine cells, secretory cells, SLC16A7+ cells and T cells) were obtained. Figure 2B is the umap diagram of the distribution of these 11 subgroups.

**TABLE1:** Subgroups define information.

cell_type	seraut_cluster
Lung epithelial cell	0
SLC16A7+ cell	1
Secretory cell	2
Endothelial cell	3
Dendritic cell	4
Secretory cell	5
Secretory cell	6
Fibroblast	7
Ionocyte cell	8
Endothelial cell	9
Ciliated cell	10
Endothelial cell	11
Secretory cell	12
T cell	13
Ionocyte cell	14
Macrophage	15
Lung epithelial cell	16
Ionocyte cell	17
Macrophage	18
SLC16A7+ cell	19
Fibroblast	20
Secretory cell	21
Neuroendocrine cell	22

### Cell trajectory analysis of single-cell subsets in LUAD

A total of 11 subpopulations were obtained through cell annotation, which were counted according to the method. The statistical results are shown in Table 2. On the premise of *p* < 0.05, five subpopulations of lung epithelial cells, macrophages, neuroendocrine cells, secret cells and T cells were highly enriched in LUAD tissues. We used Monocle to predict the developmental trajectories of five subpopulations.

**TABLE2:** Cell subpopulation statistics.

cell_name	T_celltype	T_no_celltype	N_celltype	N_no_celltype	p.val	fc
Ciliated cell	94	3,439	134	4,503	0.542639269	0.918528902
Dendritic cell	316	3,217	188	4,449	1.87E-19	2.324559025
Endothelial cell	232	3,301	842	3,795	9.42E-57	0.316768618
Fibroblast	275	3,258	175	4,462	5.70E-15	2.152152942
Ionocyte cell	229	3,304	331	4,306	0.250804955	0.901657242
Lung epithelial cell	119	3,414	1,647	2,990	7.36318877704002e-318	0.063279208
Macrophage	30	3,503	183	4,454	3.41E-20	0.208439604
Neuroendocrine cell	60	3,473	6	4,631	4.15E-16	13.33429312
Secretory cell	1,466	2067	424	4,213	4.41E-264	7.047240555
SLC16A7+ cell	694	2,839	575	4,062	5.17E-19	1.726895876
T cell	18	3,515	132	4,505	9.57E-17	0.174770464

In previous studies, LUAD tumor samples contained 18.2% tumor cells and 53.4% T cells, while normal samples contained 10.4% epithelial cells and 44.1% T cells, indicating that T cells are the dominant cell type in tumor and normal samples. Tumor-associated macrophages have strong plasticity and, if reprogrammed, can clear tumor cells and regulate the adaptive immune system for cancer immunotherapy. These studies suggested that the five cell subpopulations obtained from our analysis are potentially valuable in identifying tumors from normal tissue and in tumor treatment.

First, pseudotime was used to measure the degree of cell differentiation (Figure 2C). Next, we show the trajectory of differentiation of the five subpopulations (Figure 2E). The five subpopulations could be divided into five branches and states (Figure 2D).

The seraut_cluster of the four subgroup trajectory diagrams. Figure 2F shows that lung epithelial cells were mainly in the 0 and 16 subgroups. In the trajectory diagram, these two subgroups are on the branches of state 4 and state 5. In these subgroups, macrostat1 and macrostat15 were mainly enriched. Neuroendocrine cells were mainly enriched in subgroup 22, which was mainly on the branch of State 2 in the trajectory diagram. Secret cells were mainly enriched in subgroups 2, 5, 6, 12 and 21. In the trajectory diagram, 2 and 12 are mainly on the branches of state 4, states 5, 5 and 6 are mainly on the branches of state 1 and state 2, and 21 is mainly on the branch of state 2. T cells were mainly enriched in subgroup 13. In the trajectory diagram, 13 is mainly on the State2 branch (Supplementary Figure S4A).

**FIGURE 3:**
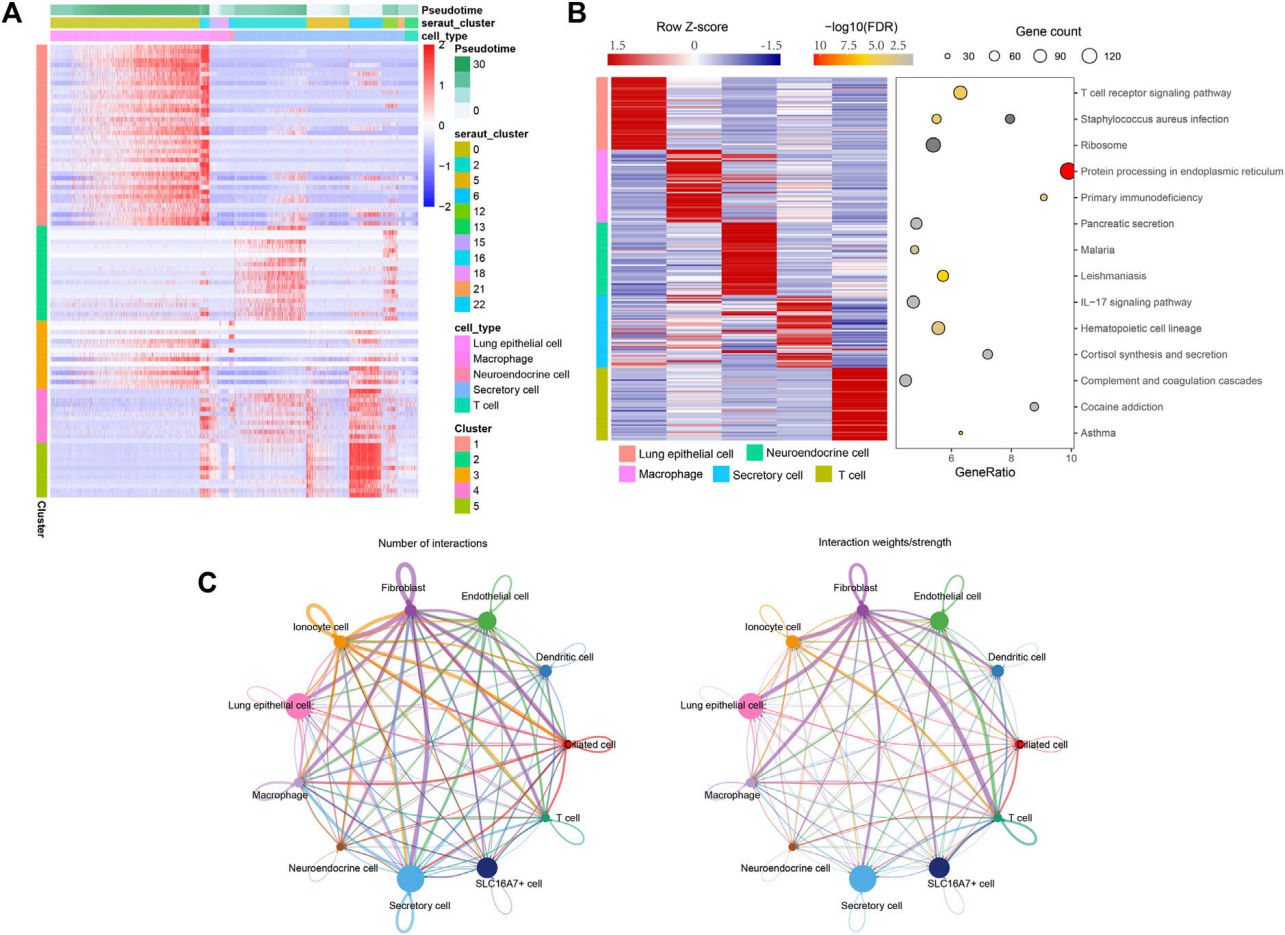
The regulatory genes of five branches. **(A)** Pedigree heatmap of 100 genes. **(B)** Enrichment analysis results of five key subgroups. Left: Expression map of the first 50 specifically expressed genes in each cell type. The value of each gene is a Z score scaled by row. Right: A representative KEGG pathway. **(C)** The interaction of cell subsets predicts that the thickness of the line is the change in the number and intensity of ligand‒receptor interactions.

Then, we used branched expression analysis modelling (BEAM) to find the regulatory genes of five branches, screened the key genes with qval (corrected P), and screened the 100 genes with the smallest qval. Therefore, we used these 100 genes to draw the pedigree heatmap (of which 100 genes are listed as cells) (Figure 3A). Information on these 100 genes is shown in Supplementary Table S3.

### Pathway enrichment of subpopulations

To further study the functions of these five subpopulations, we extracted the marker genes of these five subpopulations, conducted KEGG enrichment analysis through the webgestalt package, and screened the key pathways with FDR <0.05. The enrichment results are shown in Supplementary Table S4. The first three pathways were screened by enrichment ratio, the first 50 marker genes were screened for each subgroup, and the expression heatmap was drawn (Figure 3B).

### Communication analysis of cell subsets

Cell communication of 11-cell subsets was analysed by CellChart. Supplementary Table S5 shows the results of the cell communication analysis. From Figure 3C, it can be seen that the interaction of these 11 subsets changes in the number and intensity of ligand‒receptor interactions.

**FIGURE 4:**
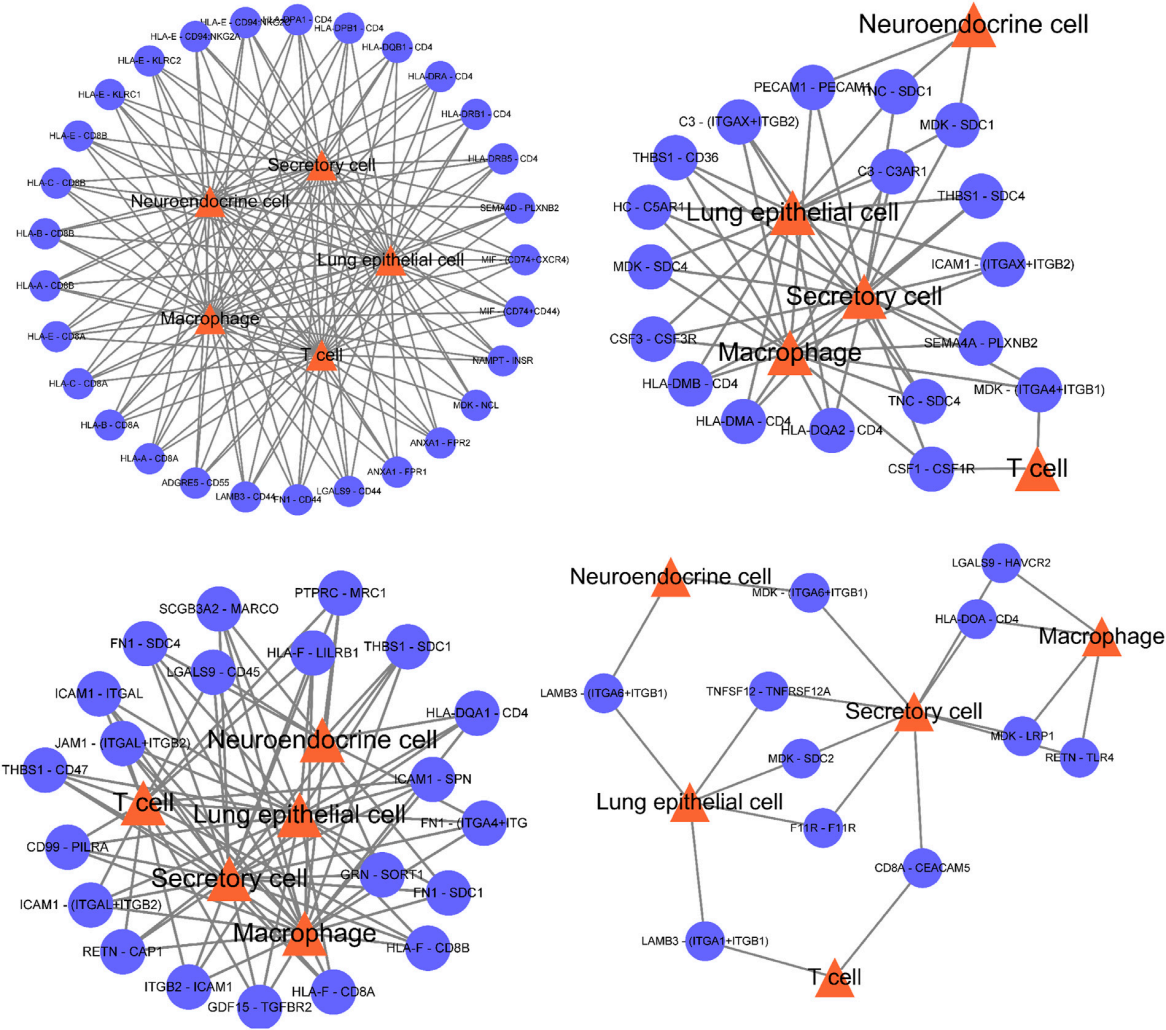
The cell communication of these five subpopulations is very complex, and there are many ligand receptors involved, which also shows that the changes in the body microenvironment are very complex in the process of tumor occurrence and development.

The predicted ligand receptor interactions of the five important subpopulations screened above were used to draw the interaction network between cell subpopulations and ligand receptors. Figure 4 shows that the cell communication of these five subpopulations is very complex, and many ligand receptors are involved, which also shows that the changes in the body microenvironment are very complex in the process of tumor occurrence and development. These five subpopulations may all play a very important role in tumorigenesis and development.

### Screening of key genes and construction and validation of prognostic risk model

The expression profile data of FPKM of TCGA were downloaded and further converted into TPM. First, we filtered through the standard deviation of the expression of each gene in all samples greater than 0.5, used the limma package to analyse the difference in the expression profile matrix of LUAD, and screened the differentially expressed genes with | logfc | > 1 and FDR <0.05. A total of 2,812 differentially expressed genes were screened, of which 1,110 genes were upregulated and 1702 genes were downregulated. Supplementary Table S6 shows the results of all gene difference analyses. Through overlap analysis, we found that 462 differentially expressed genes were marker genes of these five subgroups (Supplementary Figure S4B).

Using the expression profile data of TCGA, for the relevant genes and survival data, the R package survival coxph was used to carry out the univariate Cox proportional hazards regression model, and *p* < 0.01 was selected as the threshold for filtering. Finally, there were 16 genes with differences. The univariate Cox analysis results are shown in Supplementary Table S7.

**FIGURE 5:**
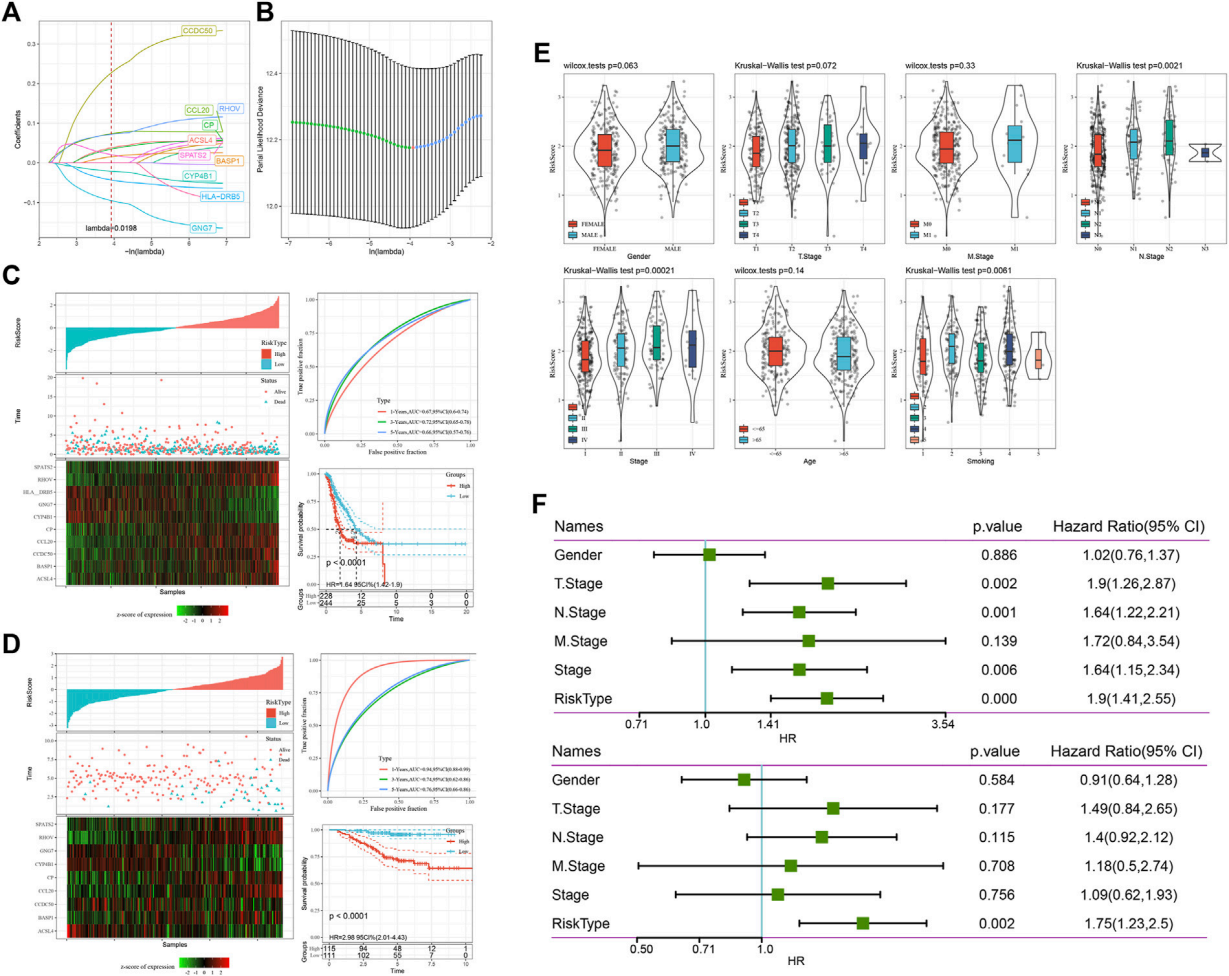
The risk model constructed and evaluated. **(A)** The change track of each independent variable. The horizontal axis represents the log value of the independent variable lambda, and the vertical axis represents the coefficient of the independent variable. **(B)** Confidence intervals under each lambda. **(C)** Risk score, survival time, survival status and 10-gene expression in TCGA. ROC curve and AUC classified by the 10-gene signature. KM survival curve distribution of the 10-gene signature. The model has a high AUC offline area, and patients with a higher risk score had a poorer prognosis. **(D)** Risk score, survival time, survival status and expression of 10 genes in GSE31210; ROC curve and AUC classified by the 10-gene signature. KM survival curve distribution of the 10-gene signature. **(E)** Comparison of the distribution of the risk score of TCGA among clinical feature groups. We found that there were significant differences among N stage, stage and smoking. **(F)** Univariate Cox regression analysis found that the risk score was significantly correlated with survival, and the corresponding multivariate Cox regression analysis found that risk type was still significantly correlated with survival.

At present, 16 genes related to prognosis in TCGA have been identified, but the large number of these genes is not conducive to clinical detection, so we need to further narrow the range of immune genes under the condition of maintaining high accuracy. We further compressed these 16 genes using lasso regression to reduce the number of genes in the risk model. First, we analysed the change trajectory of each independent variable. It can be seen that with the gradual increase in lambda, the number of independent variable coefficients tending to 0 also gradually increases. We used 10-fold cross validation to build the model. Analyse the confidence interval under each lambda. Figure 5A,B shows that when lambda = 0.0198, the model reached the optimum. Therefore, we selected 10 genes when lambda = 0.0198 as the target genes in the next step.

Finally, we obtained 10 genes: *CCL20, CP, HLA-DRB5, RHOV, CYP4B1, BASP1, ACSL4, GNG7, CCDC50, and SPATS2*. The final 10-gene signature formula is as follows:

$$\begin{array}{l} \text{RiskScore}=\text{0}\text{.}089\text{*CCL}20+\text{0}\text{.}054\text{*CP}-\text{0}\text{.}058\text{*HLA}-\text{DRB5}+\text{0}\text{.}084\text{*RHOV}-\text{0}\text{.}029\text{*}\\ \text{CYP}4\text{B}1+\text{0}\text{.}027\text{*BASP}1-\text{0}\text{.}055\text{*ACSL}4-\text{0}\text{.}118\text{*GNG}7+\text{0}\text{.}306\text{*CCDC}50+\text{ 0}\text{.}002\text{*SPATS}2 \end{array}$$

We calculated the risk score of each sample according to the expression level of the sample and drew the risk score distribution of the sample (Figure 5C). We used the R software package timeroc to analyse the ROC curve of the prognostic classification of the risk score. We analysed the classification efficiency of prognosis prediction at 1, 3, and 5 years, from which we can see that the model has a high AUC offline area. Finally, we calculated the z score for the risk score, divided the samples with risk scores greater than zero into a high-risk group and a low-risk group, and drew Kaplan‒Meier survival curves, from which we can see that patients with a higher risk score had a poorer prognosis (*p* < 0.0001).

To better evaluate the risk model constructed in this study, we used GSE31210 for verification. We calculated the risk score of each sample according to the expression level of the sample and drew the risk score distribution of the sample (Figure 5D). Similarly, we used the R software package timeROC to analyse the ROC of prognosis classification of risk score. We analysed the classification efficiency of prognosis prediction at 1, 3, and 5 years, from which we can see that the model has a high AUC offline area. Finally, we calculated the z score for the risk score, divided the samples with risk scores greater than zero into a high-risk group and a low-risk group, and drew a KM curve, from which we can see that there was a very significant difference between them (*p* < 0.0001).

### The risk score suggests that LUAD is related to smoking

By comparing the distribution of the risk score of TCGA among clinical feature groups, we found that there were significant differences among N stage, stage and smoking (*p* < 0.05) (Figure 5E).

### Univariate and multivariate analysis of the 10-gene signature

To identify the independence of the 10-gene signature risk model in clinical application, we used univariate and multivariate Cox regression to analyse the relevant HR, 95% CI of HR and *p* value in the clinical information carried by all TCGA data. We systematically analysed the clinical information recorded by TCGA patients, including sex, stage and risk type. In the TCGA datasets, univariate Cox regression analysis found that the risk score was significantly correlated with survival, and the corresponding multivariate Cox regression analysis found that risk type (HR = 1.75, 95% CI = 1.23–2.5, *p* < 0.05) was still significantly correlated with survival (Figure 5F). The above situation shows that our model 10-gene signature risk model has good prediction performance in clinical application value.

### Relationship between risk score and channel

**FIGURE 6:**
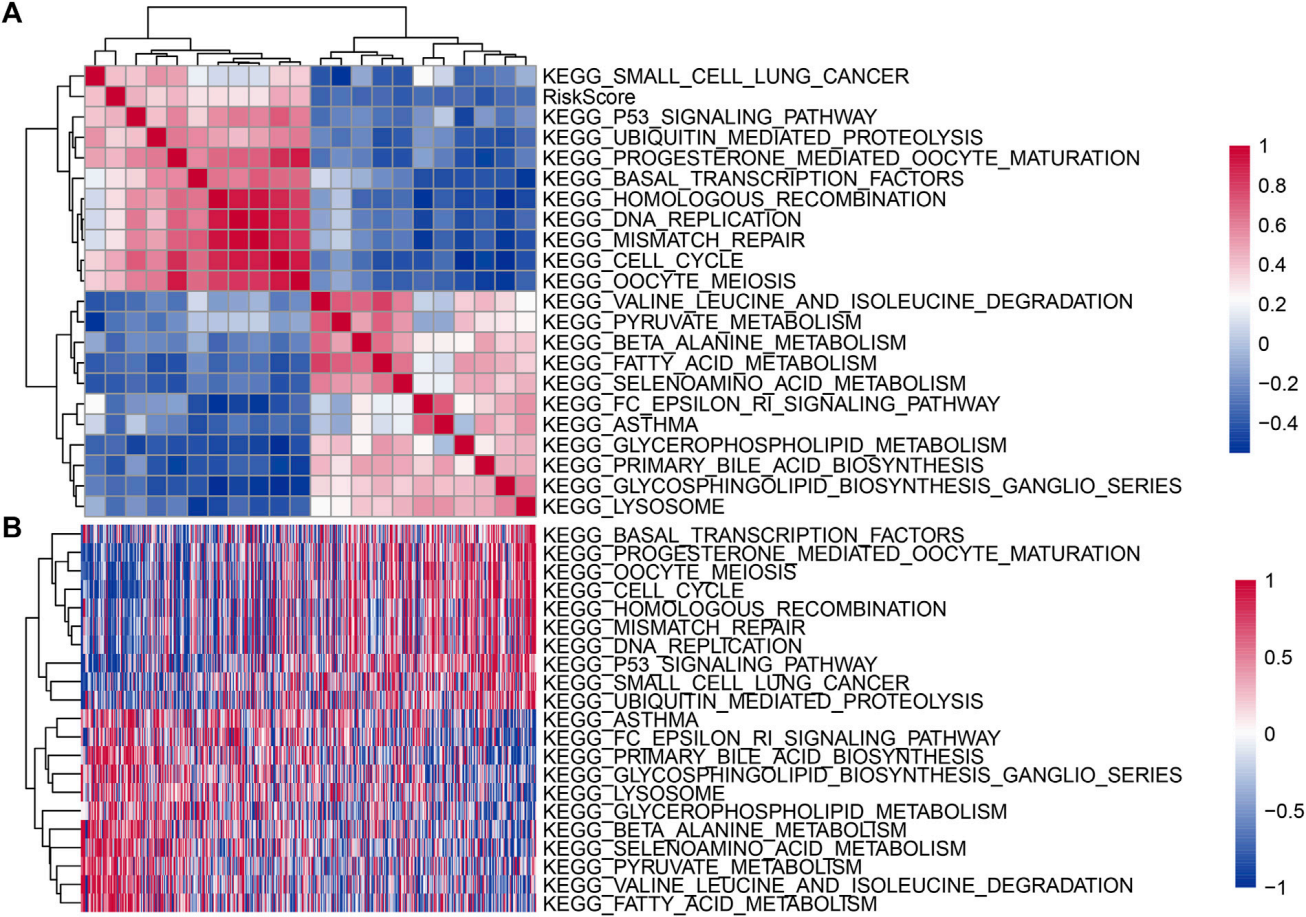
**(A)** Clustering of correlation coefficients between KEGG pathways and risk score with a correlation greater than 0.4. **(B)** For KEGG pathways with a correlation greater than 0.4 with the risk score, the change relationship of the ssGSEA score in each sample with the increase in risk score. The horizontal axis represents the sample, and the risk score increases from left to right.

To further observe the relationship between the risk scores of different samples and biological functions, we used the R software package GSVA for single-sample GSEA of the expression profile corresponding to TCGA samples, calculated the scores of each sample on different functions, obtained the ssGSEA score of each sample corresponding to each function (Supplementary Table S8), further calculated the correlation between these functions and the risk score (Supplementary Table S9), and selected the function with a correlation no less than 0.3, as shown in Figure 6A. It can be seen that 11 are negatively correlated with the sample risk score, and 10 channels are positively correlated with the sample risk score. KEGG P53 SIGNALING PATHWAY, KEGG_CELL CYCLE and KEGG_OOCYTE MEIOSIS were positively correlated with the risk score. KEGG_VALINE LEUCINE AND ISOLEUCINE DEGRADATION and KEGG FATTY ACID METABOLISM were negatively correlated with the risk score. Twenty-two KEGG pathways with correlations no less than 0.3 were identified, and cluster analysis was performed according to their enrichment scores (Figure 6B).

### Expression of the unreported signature genes HLA-DRB5 and CCDC50 in LUAD

CCL20, CP, RHOV, CYP4B1, BASP1, ACSL4, GNG7 and SPATS2 have been reported, and their dysregulation is associated with the prognosis of LUAD. However, the expression and function of HLA-DRB5 and CCDC50 have not yet been reported.

**FIGURE 7:**
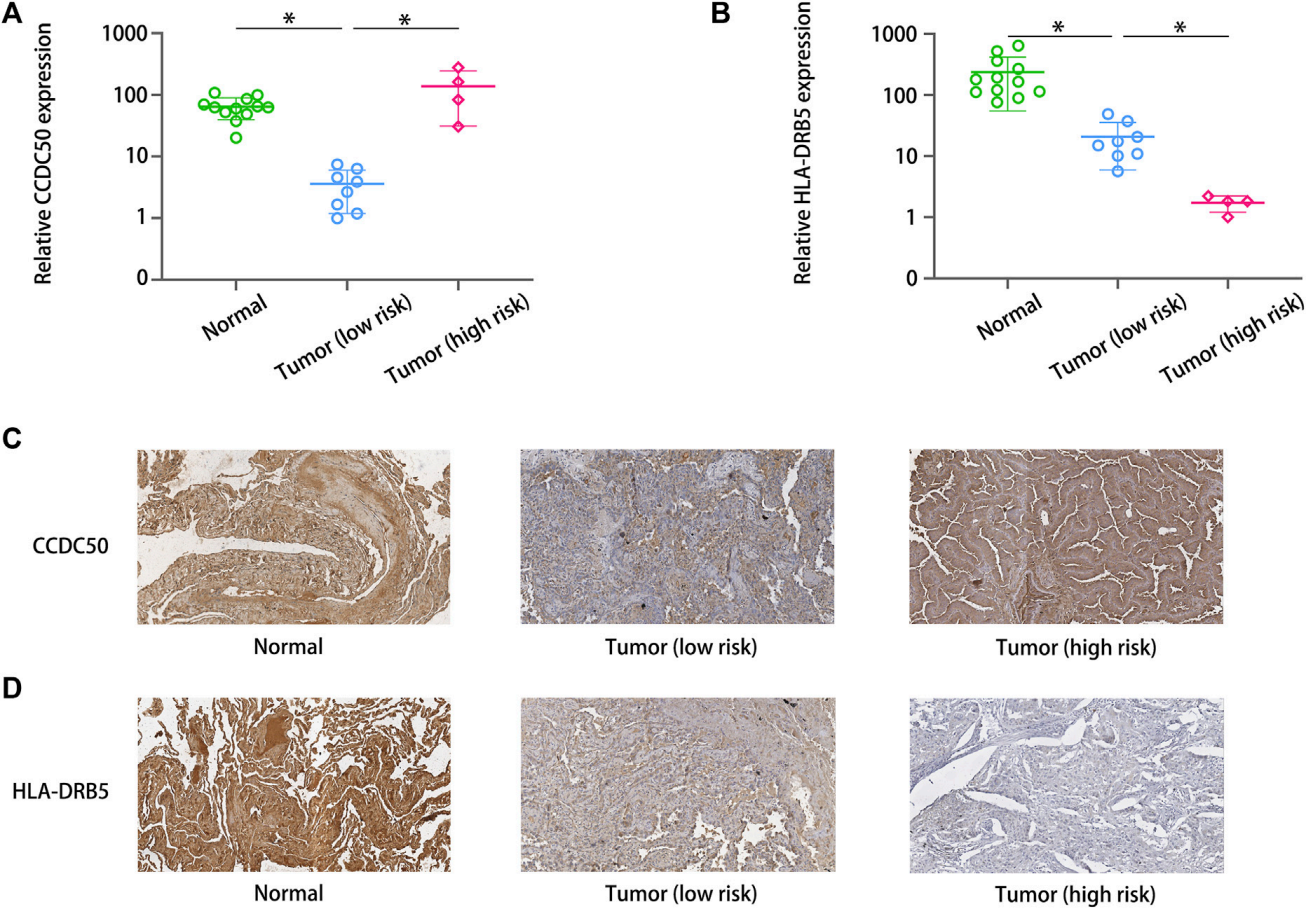
Expression of the unreported signature genes. The qRT‒PCR results revealed that the levels of **(A)** HLA-DRB5 were lower and the levels of **(B)** CCDC50 were higher in five high-risk tumor tissues. The protein levels of **(C)** HLA-DRB5 were lower and the levels of CCDC50 **(D)** were higher in high-risk tumor tissues.

We applied immunohistochemistry and qRT‒PCR to detect the differences in the expression of HLA-DRB5 and CCDC50 between paired tumor tissues and normal tissues. The qRT‒PCR results revealed that the levels of HLA-DRB5 were lower and the levels of CCDC50 were higher in five high-risk tumor tissues (Figure 7A). The protein (Figure 7C) levels of HLA-DRB5 were lower and the levels of CCDC50 (Figures 7B,D) were higher in high-risk tumor tissues.

## Discussion

At present, most studies on LUAD focus on the transcriptome level, and there are few studies on the single-cell level and tumor microenvironment (Guo et al., 2018; He et al., 2021; Spella and Stathopoulos, 2021). The heterogeneity between and within tumors is closely related to tumor progression and metastasis and will affect the response to targeted therapy and the final survival results (Chen Z. et al., 2021; Spella and Stathopoulos, 2021). Therefore, it is necessary to screen marker genes related to prognosis at the single-cell level of LUAD and construct a risk model accordingly.

First, we obtained bulkRNA-seq data for LUAD samples based on the TCGA database and downloaded the single-cell sequencing data GSE149655 from the GEO database. A total of four samples were detected, including two LUAD samples and two normal samples. The single-cell dataset GSE149655 was subjected to quality control, filtration and dimensionality reduction through the seraut package, and finally, 23 subsets were screened. Next, 23 subpopulations were annotated by the marker gene of cellmarkers, and a total of 11-cell types were annotated by 23 subpopulations. Through statistical analysis of 11 subpopulations of LUAD and normal samples, five important subpopulations were selected, namely, lung epithelial cells, macrophages, neuroendocrine cells, secret cells and T cells. Next, we screened the differentially expressed genes in TCGA-LUAD, constructed a prognostic risk model based on key genes by using univariate risk analysis and multivariate risk analysis, and verified it with the independent GSE31210 dataset. Finally, we verified the genes in the model through experiments. The above situation shows that our model 10 gene signature has good prediction performance in clinical application value.

A growing number of studies have shown that cancer usually becomes more heterogeneous in the process of disease. Due to this heterogeneity, large tumors may include a variety of cell collections, which have different molecular characteristics and different sensitivities to treatment. This heterogeneity may lead to an uneven distribution of tumor cell subsets with different genes between and within the disease site (spatial heterogeneity) or temporal changes in the molecular composition of cancer cells (temporal heterogeneity) (Dagogo-Jack and Shaw, 2018). ScRNA-seq can reveal the expression of all genes in the whole genome at the single-cell level and can study cell heterogeneity more intuitively (Navin et al., 2011; Francis et al., 2014). We screened the cell types with significant differences in subpopulation abundance between LUAD and normal tissues through single-cell analysis, screened the cell types with different subpopulation abundance during the occurrence and development of LUAD at the single-cell level, screened the marker genes of these key cell types, combined with TCGA data, screened the marker genes related to prognosis, and constructed the risk model accordingly.

The model we constructed includes 10 genes, including *CCL20, CP, HLA-DRB5, RHOV, CYP4B1, BASP1, ACSL4, GNG7, CCDC50, and SPATS2*. *CCL20* is a member of the chemokine family (Chen et al., 2020). Recent studies have shown that high levels of *CCL20* are associated with malignancies of various cancers (Kapur et al., 2016; Zhang et al., 2016). CCL20 can also recruit immune cells, such as DCs and Tregs, which further connect CCL20 with the tumor microenvironment. CCL20 upregulation can recruit CD8^+^ T cells to the immune microenvironment of LUAD, which is helpful for immunotherapy (Luo et al., 2017; Lyu et al., 2019; Ma et al., 2022). CP (ceruloplasmin) is a multi copper oxidase and a mammalian plasma ferrous oxidase (Hellman and Gitlin, 2002). Recent evidence suggests that ceruloplasmin is also associated with tumor development and progression. The expression of plasma ceruloplasmin in LUAD is significantly upregulated and significantly correlated with clinicopathological stage (Matsuoka et al., 2018). The expression of plasma ceruloplasmin was also significantly upregulated in high-grade clear cell renal cell carcinoma samples (Thibodeau et al., 2016). HLA-DRB5, whose expression products play a central role in the immune system by presenting peptides derived from extracellular proteins (Su et al., 2021). Studies have shown that HLA-DRB5 is associated with risk factors for cervical cancer (Bao et al., 2018). HLA-DRB5 was expressed at low levels in all patients with multiple myeloma, in a subgroup of patients with ulcerative mucositis and in a control group (Marcussen et al., 2017). RHOV has been shown to promote cell differentiation and act as an important regulator of neural crest induction (Guémar et al., 2007; Song et al., 2015). RHOV is highly expressed in many lung cancer cell lines and promotes the growth and metastasis of LUAD cells (Zhang Y. et al., 2020; Chen H. et al., 2021; Zhang et al., 2021). CYP4B1 belongs to the mammalian CYP4 enzyme family and is mainly expressed in human lungs (Wiek et al., 2015). Studies have suggested that CYP4B1 is a prognostic biomarker and potential therapeutic target of LUAD and can also be used as a target of cancer treatment (Lim et al., 2020; Liu et al., 2021). BASP1 can regulate many types of cell biological behavior, including proliferation, apoptosis and differentiation (Sanchez-Niño et al., 2010; Tang et al., 2017). High expression of BASP1 is associated with poor prognosis of human LUAD and head and neck squamous cell carcinoma and promotes tumor progression (Jaikumarr Ram et al., 2020; Wang et al., 2021). ACSL4 is mainly located in mitochondria, peroxisomes and the endoplasmic reticulum and plays a crucial regulatory role in ferroptosis (Quan et al., 2021). In most cases, ACSL4 plays a carcinogenic role. The high expression of ACSL4 indicates that the prognosis of patients with ovarian cancer is poor. In LUAD, ACSL4 plays a tumor suppressor role by inhibiting tumor survival/invasion and promoting ferroptosis (Ye et al., 2016; Ma et al., 2021; Yang et al., 2021). GNG7 belongs to the large G protein *γ* family (Shibata et al., 1998). Many studies have shown that GNG7 is a tumor suppressor gene in squamous cell carcinoma, pancreatic cancer, esophageal cancer, gastrointestinal cancer and clear cell renal cell carcinoma. In LUAD, GNG7 is significantly downregulated in LUAD tissues and cell lines. Low expression of GNG7 is related to poor prognosis in LUAD patients, and GNG7 overexpression inhibits the proliferation and invasion of LUAD cells. (Shibata et al., 1999; Demokan et al., 2013; Liu et al., 2019; Xu et al., 2019; Fang et al., 2022). CCDC50 is a tyrosine phosphorylated protein that mediates apoptosis through the NF-κB pathway (Bohgaki et al., 2008). However, research on CCDC50 in cancer is still insufficient. Some studies have shown that different splice variants of CCDC50 play opposite tumorigenic roles *in vitro* and *in vivo*. CCDC50-S promotes the metastasis of renal clear cell carcinoma, but CCDC50-FL and sh-CCDC50 inhibit the metastasis of renal clear cell carcinoma (Sun et al., 2020). SPATS2 is a cytoplasmic RNA-binding protein that plays an important role in spermatogenesis (Fang et al., 2022). In recent studies, the expression of SPATS2 was upregulated in HCC tissues. High expression of SPATS2 was associated with poor clinicopathological features and poor prognosis in HCC patients. SPATS2 knockdown significantly inhibited the growth and invasion of HCC cells and promoted apoptosis and G1 arrest of HCC cells *in vitro* (Senoo et al., 2002). SPATS2 is also highly expressed in liver cancer and may be a new diagnostic and prognostic biomarker of liver cancer. In recent studies, SPATS2 has also been used as a diagnostic biomarker of LUAD (Dong et al., 2020; Xing et al., 2020). The above reports of gene dysregulation associated with LUAD are consistent with our risk gene prediction results, which showed that the 10-gene signature can be used as an effective prognostic tool for LUAD patients. However, there are still some deficiencies: 1. We need to use more clinical samples for further verification in the follow-up. The biological functions of newly discovered HLA-DRB5 and CCDC50 risk genes in lung cancer were further explored.

## Conclusion

By analysing the single-cell sequencing data of LUAD, we established a 10-gene signature related to the prognosis of LUAD. This 10-gene signature has strong robustness and can achieve stable prediction efficiency in datasets from different platforms. We also performed qPCR and immunohistochemical sample verification on CCDC50 and HLA-DRB5, two genes that have not been verified in LUAD. The results are consistent with our prediction. These findings will contribute to a more accurate diagnosis of LUAD, which is very important for the precise treatment of LUAD.

## Data Availability

The datasets presented in this study can be found in online repositories. The names of the repository/repositories and accession number(s) can be found in the article/Supplementary Material.

## Supplementary material

Supplementary Figure S1 Quality control chart (before filtration) of LUAD single-cell subsets.

Supplementary Figure S2 Quality control chart (after filtration) of LUAD single-cell subsets.

Supplementary Figure S3 Number of cells in each sample before (A) and (B) after filtration. (C) PCA dimensionality reduction (left) shows the distribution of hypervariable genes and nonhypervariable genes, and the top 20 hypervariable genes are shown on the right.

Supplementary Figure S4 (A) PCA dimensionality reduction to find anchor points. In the trajectory diagram, 13 is mainly on the State2 branch. (B) Among the 2812 differentially expressed genes, 462 were marker genes of these four subgroups by overlap analysis.

Supplementary Table S1 The results of marker genes.

Supplementary Table S2 Cell marker genes.

Supplementary Table S3 Information on these 100 genes.

Supplementary Table S4 The enrichment results of KEGG enrichment.

Supplementary Table S5 The results of cell communication analysis.

Supplementary Table S6 The results of all gene difference analyses.

Supplementary Table S7 The univariate Cox analysis results.

Supplementary Table S8 The ssGSEA score of each corresponding sample.

Supplementary Table S9 The correlation between these functions and the risk score.
