# Supplementary figures and images for: Single-cell RNA sequencing analysis to explore immune cell heterogeneity and novel biomarkers for the prognosis of lung adenocarcinoma
Source: Front Genet. 2022 Aug 15;13:975542. doi: 10.3389/fgene.2022.975542 (PMC9486955; doi:10.3389/fgene.2022.975542)

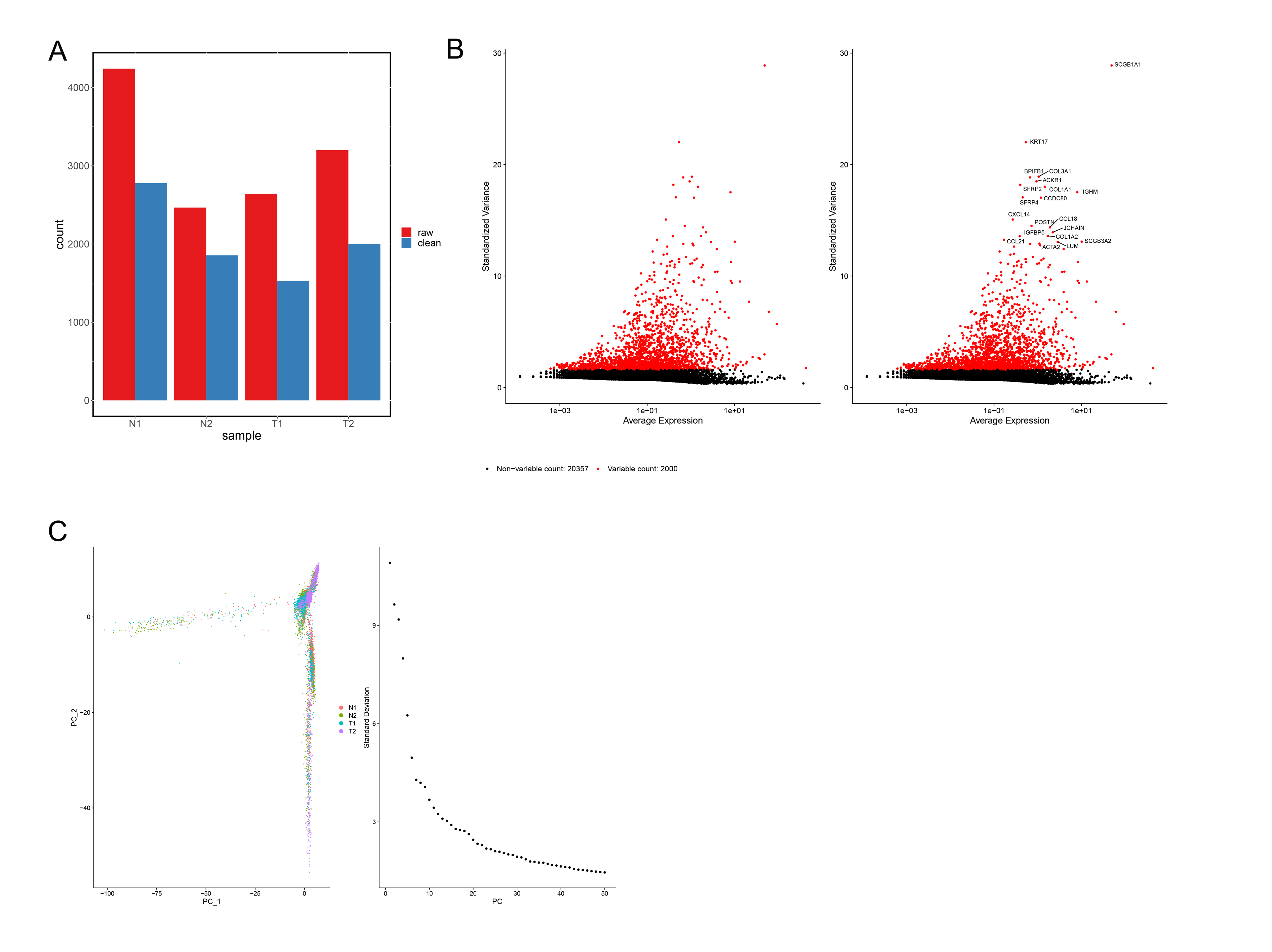

Supplement: Supplementary file 4 [file Image3.TIF]

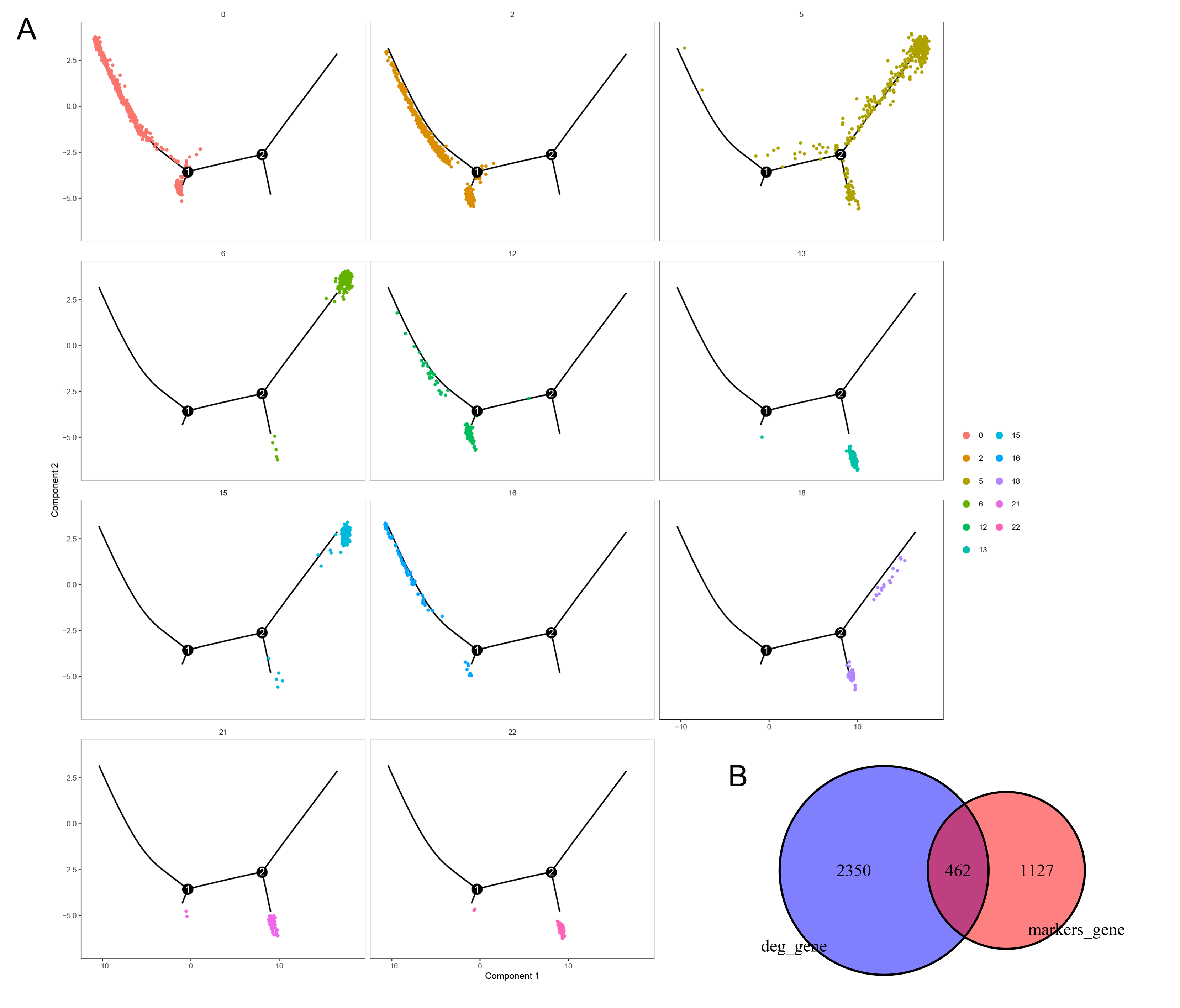

Supplement: Supplementary file 5 [file Image4.TIF]

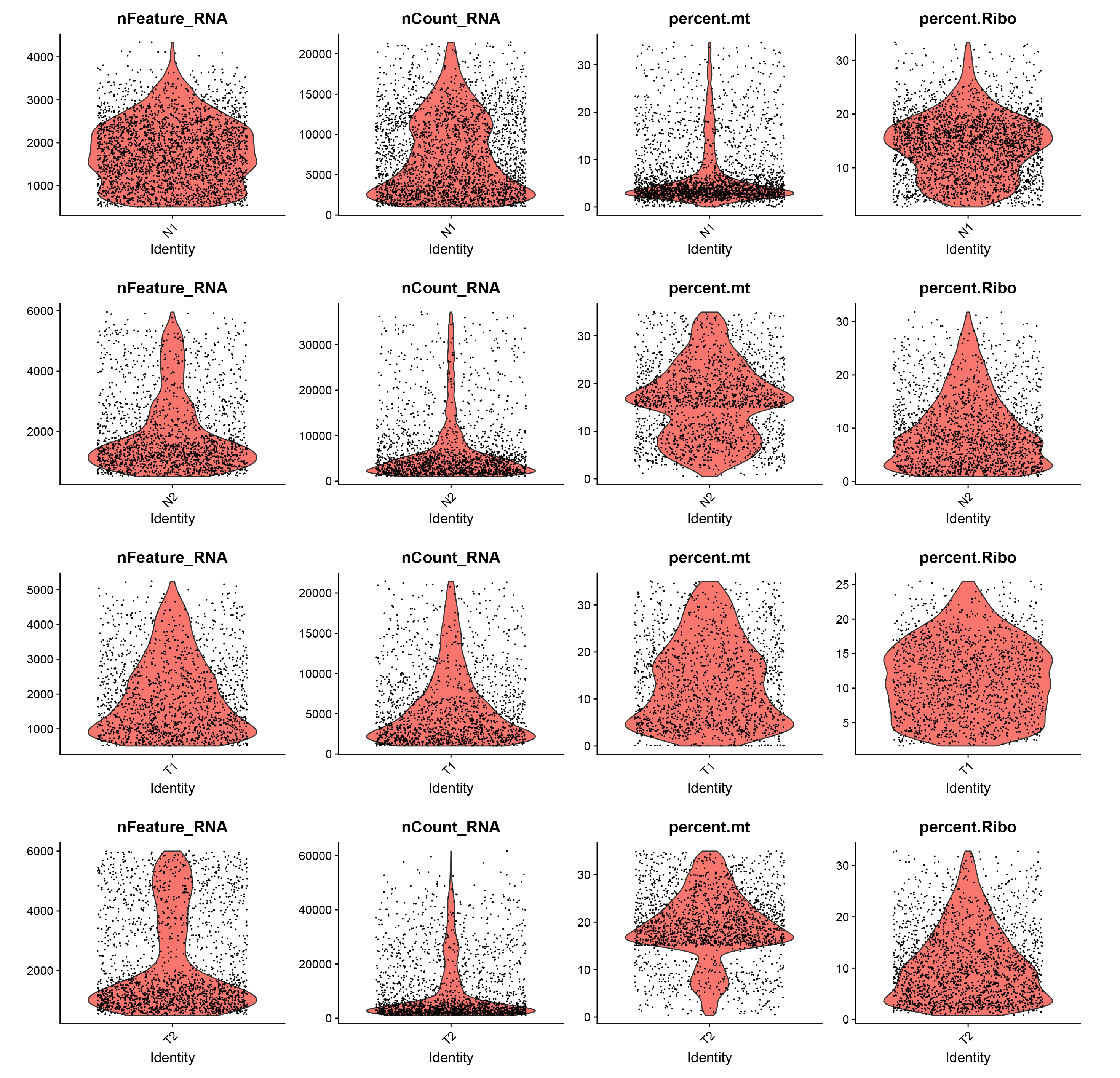

Supplement: Supplementary file 7 [file Image2.TIF]

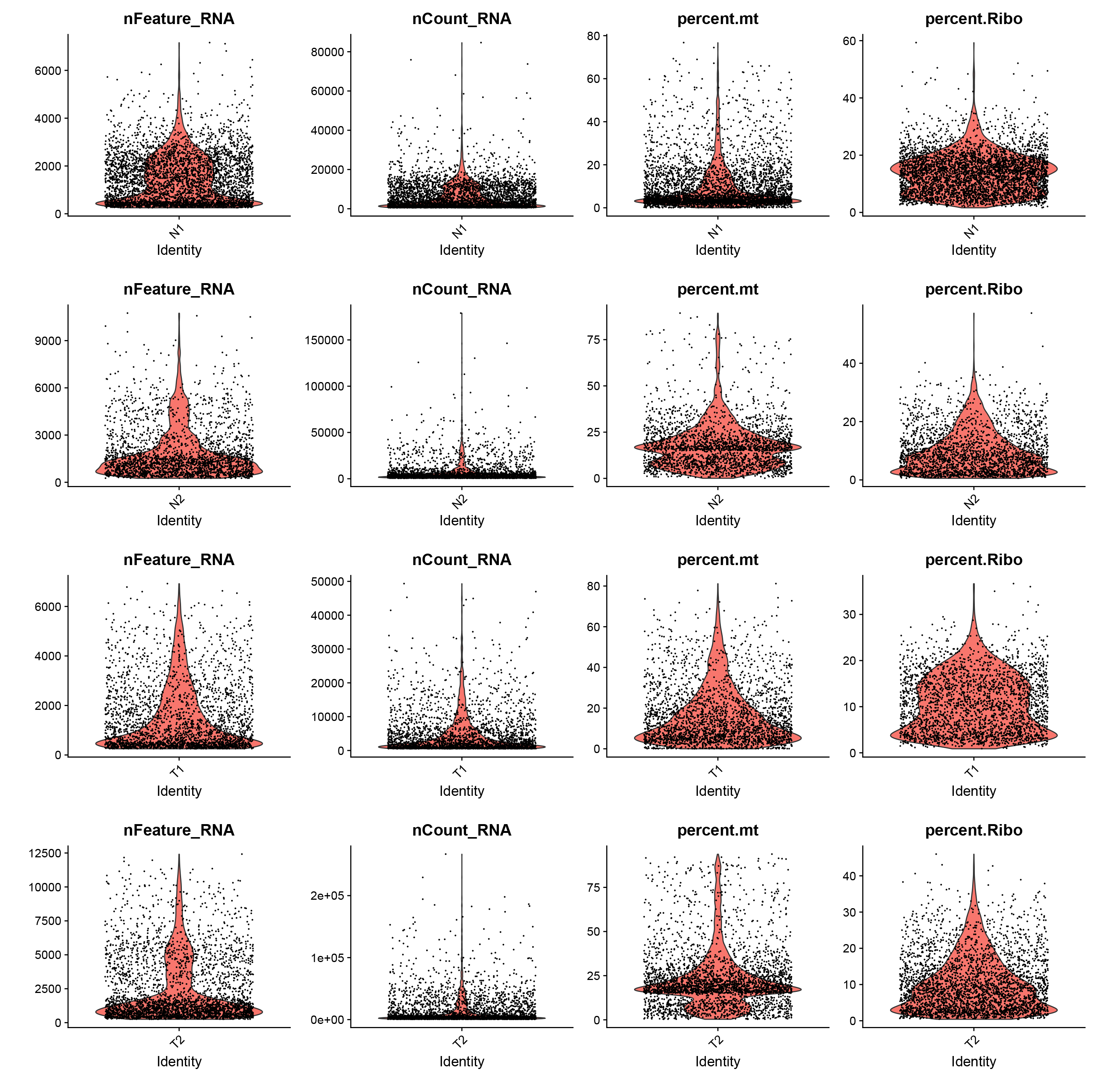

Supplement: Supplementary file 9 [file Image1.TIF]
